# Plasma Efavirenz Concentrations Are Associated With Lipid and Glucose Concentrations

**DOI:** 10.1097/MD.0000000000002385

**Published:** 2016-01-15

**Authors:** Phumla Zuleika Sinxadi, Helen Margaret McIlleron, Joel Alex Dave, Peter John Smith, Naomi Sharlene Levitt, David William Haas, Gary Maartens

**Affiliations:** From the Division of Clinical Pharmacology, Department of Medicine, University of Cape Town (PZS, HMM, PJS, GM); Division of Endocrinology and Diabetic Medicine, Department of Medicine, University of Cape Town. Cape Town, South Africa (JAD, NSL); Vanderbilt University School of Medicine, Departments of Medicine, Pharmacology, Pathology, Microbiology & Immunology (DWH); Meharry Medical College, Department of Internal Medicine, Nashville, TN, United States of America (DWH).

## Abstract

Efavirenz-based antiretroviral therapy (ART) has been associated with dyslipidemia and dysglycemia, risk factors for cardiovascular disease. However, the pathogenesis is not well understood. We characterized relationships between plasma efavirenz concentrations and lipid and glucose concentrations in HIV-infected South Africans.

Participants on efavirenz-based ART were enrolled into a cross-sectional study. The oral glucose tolerance test was performed after an overnight fast, and plasma drawn for mid-dosing interval efavirenz, fasting total cholesterol, low-density lipoprotein (LDL) and high-density lipoprotein (HDL) cholesterol, and triglycerides concentrations.

Among 106 participants (77 women), median age was 38 years, median CD4 + T-cell count was 322 cells/μL, median duration on ART was 18 months, and median (interquartile range) efavirenz concentration was 2.23 (1.66 to 4.10) μg/mL. On multivariable analyses (adjusting for age, sex, body mass index, and ART duration) doubling of efavirenz concentrations resulted in mean changes in mmol/L (95%CI) of: total cholesterol (0.40 [0.22 to 0.59]), LDL cholesterol (0.19 [0.04 to 0.30]), HDL cholesterol (0.14 [0.07 to 0.20]), triglycerides (0.17 [0.03 to 0.33]), fasting glucose (0.18 [0.03 to 0.33]), and 2-h glucose concentrations (0.33 [0.08 to 0.60]). Among 57 participants with *CYP2B6* genotype data, associations between slow metabolizer genotypes and metabolic profiles were generally consistent with those for measured efavirenz concentrations.

Higher plasma efavirenz concentrations are associated with higher plasma lipid and glucose concentrations. This may have implications for long-term cardiovascular complications of efavirenz-based ART, particularly among populations with high prevalence of *CYP2B6* slow metabolizer genotypes.

## Abstract 2

Supplemental Digital Content is available in the text

## INTRODUCTION

The non-nucleoside reverse transcriptase inhibitor (NNRTI) efavirenz is extensively prescribed and is included in the World Health Organization's preferred first-line ART regimens for HIV-1-infected adults, adolescents, and children at least 3 years of age.^1^ Efavirenz-based ART has been associated with the development of dysglycemia^2^ and dyslipidemia,^3–5^ specifically increases in total cholesterol: HDL cholesterol ratio, LDL cholesterol, and triglycerides.^6,7^ The pathogenesis of these metabolic effects are unclear, although it has been suggested that efavirenz may contribute to mitochondrial toxicity caused by concomitant thymidine analog nucleoside reverse transcriptase inhibitors (NRTI).^8^

Data are scant regarding relationships between plasma efavirenz concentrations and plasma glucose or lipid concentrations. There is considerable interindividual variability in plasma efavirenz exposure, which is largely explained by 3 *CYP2B6* loss-of-function polymorphisms.^9,10^ The 2 polymorphisms with the greatest effect, *CYP2B6* 516G→T and *CYP2B6* 983T→C, are particularly frequent with African ancestry.^11,12^ The *CYP2B6* 516G→T polymorphism is also frequent in Thai and Cambodian populations.^13,14^

We investigated whether plasma efavirenz concentrations correlated with plasma lipid and/or glucose concentrations in HIV-infected South Africans. We hypothesized that higher plasma efavirenz concentrations would be associated with higher lipid and glucose concentrations.

## MATERIALS AND METHODS

### Study Design and Participants

We conducted a prospective cross-sectional study of consecutive HIV-infected African adults who presented for routine follow-up visits at 1 community-based (Crossroads) and 1 hospital-based (Groote Schuur) ART clinic in Cape Town, South Africa. Participants were recruited by convenient sampling between February 2007 and September 2008. South African ART guidelines at the time of this study recommended an NNRTI plus 2 NRTIs (stavudine or zidovudine, each with lamivudine) as first-line therapy. Eligible participants were on efavirenz-based ART for at least 6 months. Exclusion criteria included pregnancy, renal or hepatic disease, active opportunistic infections, treatment for diabetes or dyslipidemia, and self-reported non-adherence. The study was conducted in accordance with the Declaration of Helsinki and the South African Good Clinical Practice. The University of Cape Town Research Ethics Committee approved the study (REC REF 128/2007). All participants gave written informed consent.

### Clinical and Laboratory Evaluations

Participants were instructed to fast overnight and to document the time of the evening dose of efavirenz on the day preceding the study visit. On the study day, participants underwent an oral glucose tolerance test (OGTT). Blood was drawn at 0 and 120 min after ingesting 75 g of glucose in 250 mL of water, and kept on ice until centrifuged within 4 h. Plasma for efavirenz quantification was collected into 4 mL lithium heparin tubes, kept on ice until centrifuged within 4 h, and was aliquotted and promptly frozen at −20°C, then stored at −70°C until analysis at the end of recruitment in 2008. Plasma efavirenz, fasting glucose, cholesterol, and triglyceride were quantified using the 0 min OGTT samples.

Efavirenz was quantified by a validated method using liquid chromatography-tandem mass spectrometry (LC-MS/MS) on an Applied Biosystems MDS Sciex API 4000 tandem mass spectrometer at our ISO17025 compliant and accredited analytical laboratory as previously described.^15^ The assay range of quantification was 0.05 to 20 μg/mL. Accuracy ranged from 94 to 103%. Serum glucose and lipid concentrations were determined by standard methods using the ACE Alera Clinical Chemistry System (Alfa Wassermann Diagnostic Technologies, Woerden, Netherlands). Diabetes, impaired fasting glucose, and impaired glucose tolerance were defined according to American Diabetes Association criteria.^16^ Hypercholesterolaemia, high LDL cholesterol, low or high HDL, and hypertriglyceridaemia were defined according to the NCEP III criteria.^17^

Medical records were reviewed to determine duration on ART, plasma HIV-1 RNA concentrations, and CD4 + T-cell counts. Baseline CD4 + T-cell counts were recorded, and current CD4 + T-cell count was defined as the most recent value within 3 months of enrolment. Adherence was assessed using a validated 4-day adherence questionnaire administered by trained staff.^18^

### Pharmacokinetic and Statistical Analysis

Medians (interquartile ranges) and proportions or ratios were used to describe continuous and categorical data, respectively. Scatter plots to visualize relationships between plasma efavirenz concentrations and metabolic parameters were generated using Prism version 6 (GraphPad Software, Inc., La Jolla, CA). Plasma efavirenz concentrations were log_10_ transformed to approximate normality. Associations between log_10_ transformed plasma efavirenz concentrations and cholesterol, triglycerides, and glucose were determined using univariate and multivariate linear regression analyses on Stata (version 11, StataCorp, College Station, TX). Univariate linear regression analyses characterized associations between log_10_ transformed association as an independent variable and several dependent variables: fasting total cholesterol, LDL cholesterol, HDL cholesterol, triglycerides, glucose, and 2-h OGTT glucose. Multivariate linear regression analyses adjusted for age, sex, body mass index (BMI), and total duration on ART. These potential confounders were chosen a priori. Sensitivity analyses included adjusting for current stavudine use. Case sensitivity analyses with the participant with the highest plasma concentrations were done. Missing data were not imputed.

In a subset of participants with available *CYP2B6* genotype data, we explored relationships between *CYP2B6* polymorphisms known to predict increased plasma efavirenz concentrations, *CYP2B6* 516G>T (rs3745274), 983T>C (rs28399499), and 15582C>T (rs4803419), and metabolic parameters using linear regression in PLINK.^19^ We did not correct for multiple comparisons. Genotyping was done in the Vanderbilt DNA Resources Core as described elsewhere.^20^ Composite 516/983 or 15582/516/983 genotypes were as assigned as previously described.^20^

## RESULTS

A total of 107 participants were recruited into the study. One participant with an efavirenz concentration below the limit of assay quantification was excluded from analyses for presumed nonadherence. Characteristics of the 106 evaluable participants are provided in Table 1. All participants were black Africans and most were women, reflecting the patient population at these clinics. More than 90% had been exposed to stavudine. All participants reported 100% adherence. Only 34 participants had plasma HIV-1 RNA data available within the previous 3 months. Metabolic abnormalities, as defined by NCEP III and ADA criteria, were common: hypercholesterolaemia was present in 40%, hypertriglyceridaemia in 26%, low HDL cholesterol in 49%, high LDL cholesterol in 42%, impaired fasting glucose in 24%, impaired glucose tolerance in 10%, and diabetes in 2%.

**TABLE 1:**
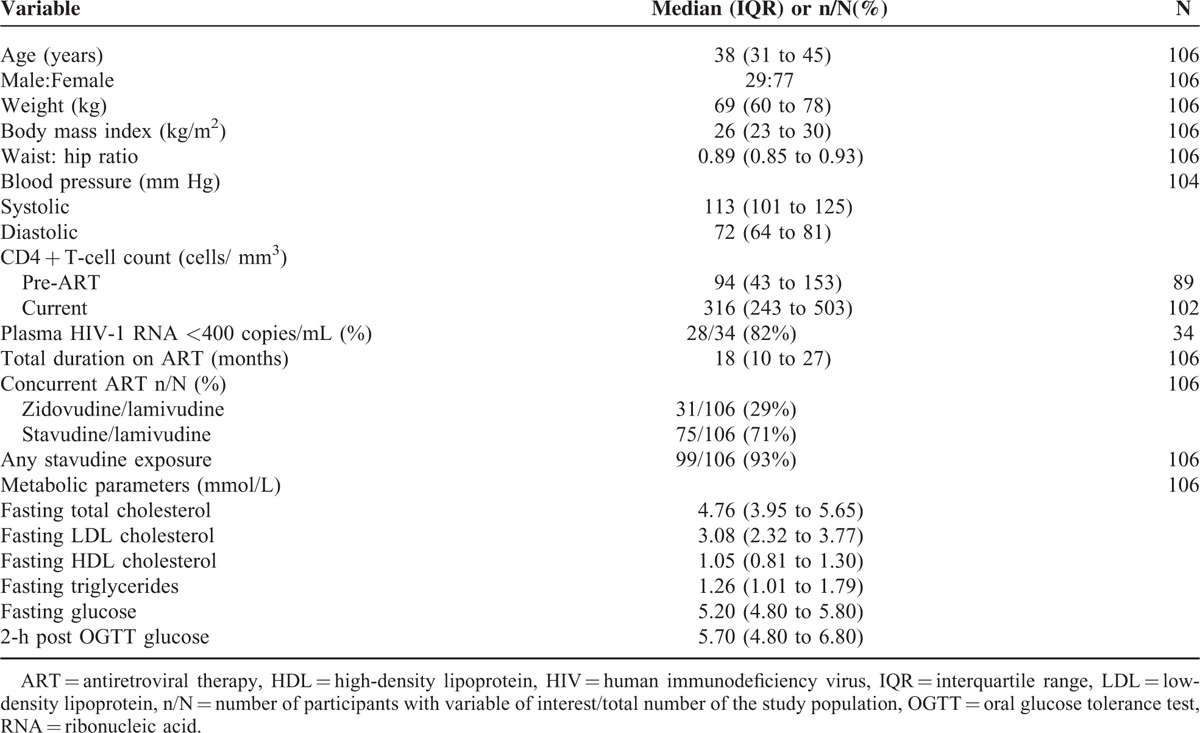
Study Participant Characteristics

**FIGURE 1:**
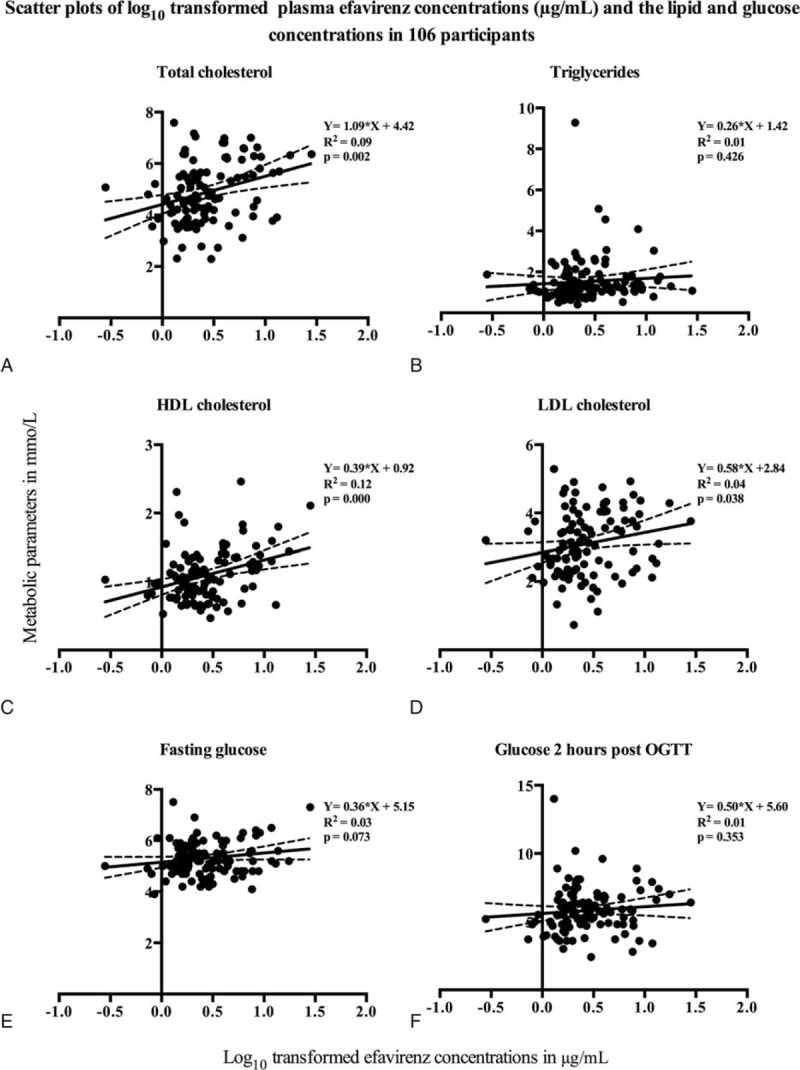
Scatter plots of log_10_ transformed plasma efavirenz concentrations (μg/mL) and the lipid and glucose concentrations in 106 participants. The *x*-axes represent log_10_ transformed plasma efavirenz concentrations. The *y*-axes represent concentrations of each metabolic parameter—(A) fasting total cholesterol, (B) fasting triglycerides, (C) fasting HDL cholesterol, (D) fasting LDL cholesterol, (E) fasting glucose, and (F) glucose 2 h post oral glucose tolerance test. Each black marker denotes an individual lipid or glucose concentration plotted against the plasma efavirenz concentrations in 106 participants. The solid black line indicates the regression line, with the 95% confidence interval shown in dotted lines. The equations for the regression line (with the slope and *y* intercept), correlation determinants (*R*^2^), and *P* values are shown on each plot. LDL = low-density lipoprotein, HDL = high-density lipoprotein.

The median efavirenz concentration was 2.23 μg/mL (IQR 1.66 to 4.10 μg/mL). Median time after the last dose was 12.08 h (IQR 11.62 to 12.78 h). Relationships between plasma efavirenz concentrations and metabolic parameters are shown in Figure 1. There was a positive correlation between higher log_10_ transformed efavirenz concentrations and higher fasting total cholesterol, HDL cholesterol, LDL cholesterol concentrations, but not with triglycerides and 2-h OGTT glucose concentrations. There was a nonsignificant correlation between plasma efavirenz concentrations and fasting glucose concentrations (*P* = 0.073).

In multivariate analyses that adjusted for age, sex, BMI, and duration on ART, log_10_ transformed efavirenz concentrations were independently associated with both lipid and glucose concentrations as displayed in Table 2. Table 2 also shows the mean change in each metabolic parameter per doubling of efavirenz concentrations. In these multivariate analyses, advancing age was also independently associated with increasing cholesterol (total cholesterol, HDL and LDL cholesterol) and glucose (fasting and 2 h), but not triglycerides (data not shown). When current stavudine use was included in the multivariate model with age, sex, BMI, and total duration on ART, similar associations between the log_10_ transformed efavirenz concentrations and both lipid and glucose concentrations were found (see Table, Supplementary Digital Content 1, a table that illustrates the multivariate regression analyses adjusting for age, sex, BMI, total duration on ART, and current stavudine use). Current stavudine use was not included in the final model because it was not a significant variable in all models (data not shown).

**TABLE 2:**
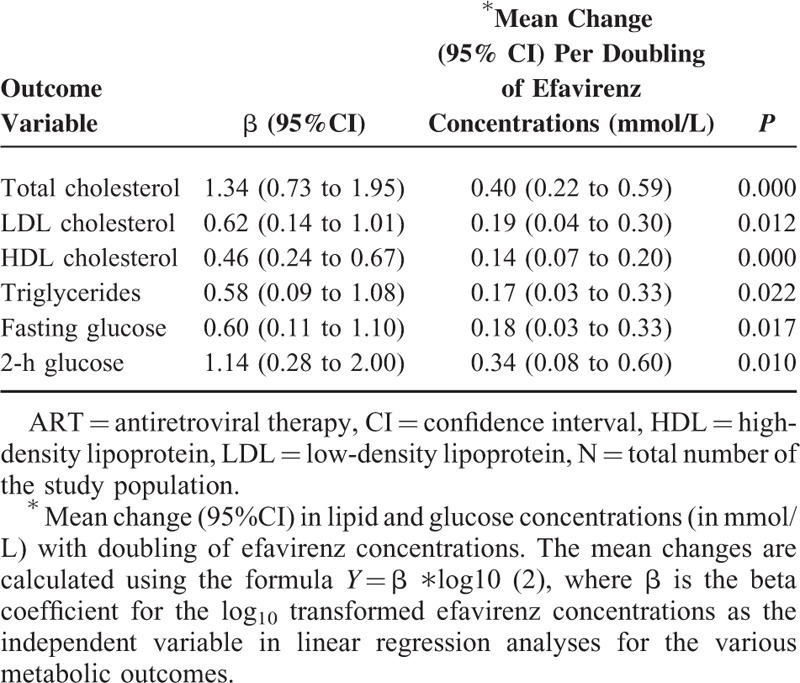
Multivariate Linear Regression Analyses (Each Adjusted for Age, Body Mass Index, and Total Duration on ART) Between log_10_ Transformed Efavirenz Mid-Dosing Interval Concentrations and Each Metabolic Parameter (N = 106)

In the final multivariate regression model that included log_10_ transformed efavirenz concentrations, age, sex, BMI, and total duration on ART, the log_10_ transformed efavirenz concentrations explained 26% of interindividual variability for total cholesterol, 23% for HDL cholesterol, 20% for triglycerides, 19% for 2-h glucose, 17% for fasting glucose, and 16% for LDL cholesterol.

Exploratory analysis examined relationships between *CYP2B6* genotypes and metabolic parameters among 57 participants with available genotype data. Allelic frequencies of *CYP2B6* 516G→T, 983T→C, and 15582C→T were 0.35, 0.08, and 0.08, respectively. Genotype frequencies are displayed in Supplemental Digital Content 2 (see figure, Supplemental Figure 1, that illustrates a bar graph displaying *CYP2B6* genotype frequencies in 57 South African adults). There was a significant association between *CYP2B6* 516G→T and total cholesterol concentrations (*P* = 0.048) as shown in Table 3. In addition, for *CYP2B6* 516G→T, beta coefficients for total cholesterol, LDL cholesterol, HDL cholesterol, and triglycerides were generally consistent and in the same direction as beta coefficients for log_10_ plasma efavirenz concentrations for these parameters. There were trends toward significant associations between *CYP2B6* 516G→T and HDL cholesterol concentrations, and between composite 516/983 genotype and total and HDL cholesterol concentrations.

**TABLE 3:**
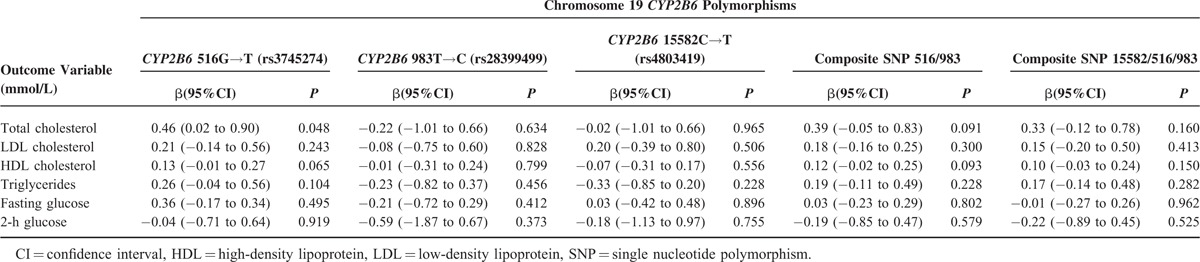
Exploratory Analyses Between Known *CYP2B6* Polymorphisms and Metabolic Parameters in 57 Participants

## DISCUSSION

We investigated whether plasma efavirenz concentrations correlated with plasma lipid and/or glucose concentrations in HIV-infected South Africans. Higher plasma efavirenz concentrations were associated with significantly higher plasma fasting lipid concentrations and higher glucose concentrations in 106 HIV-infected South African adults receiving ART in multivariate analyses. In a subset of 57 participants with available *CYP2B6* genotype data, associations between slow metabolizer genotypes and metabolic profiles were generally consistent with associations based on measured efavirenz concentrations.

To our knowledge, this is the first report of positive associations between plasma efavirenz concentrations and LDL cholesterol, or glucose concentrations. Our findings are consistent with a previous study that showed an association between plasma efavirenz concentrations and fasting HDL cholesterol in 34 participants,^21^ but disagree with a retrospective study that found no associations between plasma efavirenz concentrations and either fasting HDL cholesterol or triglycerides in 59 participants.^22^

The advent of ART has greatly reduced morbidity and mortality among HIV-infected patients.^23^ However, the D:A:D (Data Collection on Adverse Events of Anti-HIV Drugs) study showed that patients treated with protease inhibitors or NNRTIs, alone or in combination, have elevated total cholesterol, after controlling for known risk factors such as age, BMI, and sex.^24^ Some of these abnormalities, notably LDL cholesterol and glucose, are associated with an increased risk of vascular disease in the general population.^25,26^

The prevalence of metabolic complications in our cohort was high (dysglycemia 37% and dyslipidemia 47%). The commonest dyslipidemia we found was low HDL cholesterol in 49%, which is <71% we found in ART-naive patients drawn from the same clinics as the participants in our study (JA Dave, MBChB PhD, unpublished data, November 2015). However, high LDL cholesterol and high triglycerides, which were common in our study population, were rare in ART-naive patients. High prevalence of both dyslipidemia and dysglycemia in patients on ART has also been reported from other African countries.^27–29^

There is considerable interindividual variability in plasma efavirenz exposure, ∼34% of which is explained by 3 *CYP2B6* loss-of-function polymorphisms, *CYP2B6* 516G→T, 983T→C, and 15582C→T.^10,20^ Among South Africans, minor allele frequencies of *CYP2B6* 516G→T, 983T→C, and 15582C→T have been reported to be 0.36, 0.07, and 0.09, respectively.^20^ Both 516G→T and 983T→C are more frequent with African than with European ancestry. The *CYP2B6* 983T→C allele is found almost exclusively with African ancestry,^11^ and has a somewhat greater effect on efavirenz concentrations per allele than does 516G→T, whereas 15582C→T has a much lesser effect than 516G→T. The lower prevalence of *CYP2B6* slow metabolizer genotypes with European ancestry may explain, in part, why some studies conducted largely in Europeans have not shown associations between efavirenz and cardiovascular risk.^30,31^ In our study, the smaller number of participants with genotype data may have limited our ability to identify statistically significant genetic associations.

Our results have potential public health implications. Efavirenz is the preferred third drug, in combination with NRTIs, as first-line therapy in resource-limited settings where the HIV-1 burden is greatest.^1^ Efavirenz may be more likely to result in an increased risk of cardiovascular events among populations in whom *CYP2B6* slow metabolizer genotypes are prevalent. However, higher efavirenz concentrations were also associated with higher HDL cholesterol in our study, which has been associated with decreased risk of cardiovascular events. Newer agents such as rilpivirine and dolutegravir have little or no effect on lipids,^32,33^ these drugs are currently not available in most low-middle income countries.

Our study has several limitations. Because it is a cross-sectional study, we cannot compare changes from baseline in lipid or glucose concentrations in patients starting ART. The viral load data was determined by reviewing medical records and only 34 participants had viral load data recorded. Therefore, viral load was not included in our multivariate analyses. Our sample size is relatively small. We investigated associations with mid-dose interval efavirenz concentrations and metabolic parameters, but other pharmacokinetic parameters (eg area under the concentration-time curve) might better describe the relationships with metabolic profiles. Although participants recorded the time of last dose on the day before pharmacokinetic sampling, we cannot exclude incomplete adherence.

## CONCLUSIONS

In conclusion, higher plasma efavirenz concentrations were associated with higher plasma lipid and glucose concentrations. However, larger cohort studies are needed to replicate these associations. Well-powered studies in Africa and other regions where efavirenz slow metabolizer genotypes are prevalent are needed to assess whether long-term efavirenz use is associated with increased risk of cardiovascular events.

## Supplementary Material

Supplemental Digital Content
