# Supplementary material for: Plasma Efavirenz Concentrations Are Associated With Lipid and Glucose Concentrations
Source: Medicine (Baltimore). 2016 Jan 15;95(2):e2385. doi: 10.1097/MD.0000000000002385 (PMC4718242; doi:10.1097/MD.0000000000002385)
Supplement: Supplemental Digital Content [file medi-95-e2385-s001.pdf]

PLASMA EFAVIRENZ CONCENTRATIONS ARE ASSOCIATED WITH LIPID AND GLUCOSE CONCENTRATIONS

Sinxadi et al

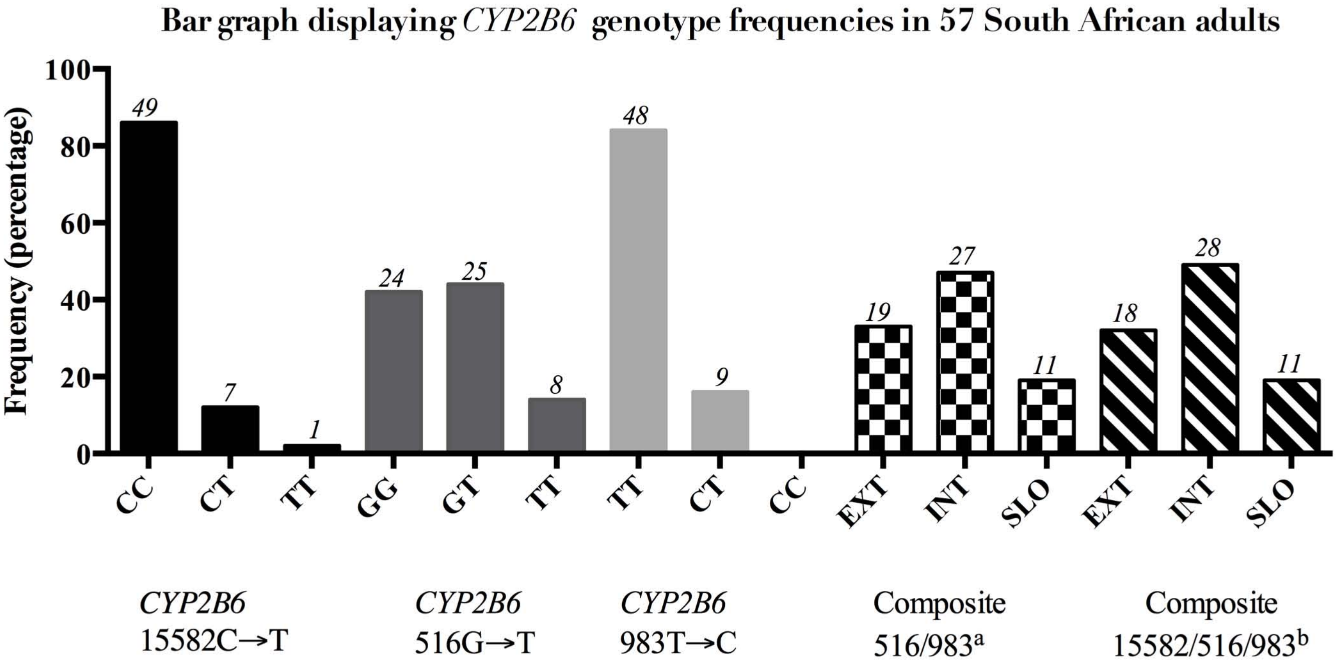

Supplemental Figure 1. **Bar graph displaying *CYP2B6* genotype frequencies in 57 South African adults.** The x-axis represents CYP2B6 genotypes: 15582C→T (solid black), *CYP2B6* 516G→T (dark grey), 983T→C (light grey), composite 516/983 (black and white squares), and composite 15582/516/983 (black and white horizontal lines) genotypes. The composite genotypes were categorized into three levels; extensive metabolizer (EXT), intermediate metabolizer (INT) and slow metabolizer (SLO). The y-axis represents genotype frequencies in percentages. The number in italics at the top of each bar displays the absolute count. a Composite 516/983 were assigned as follows: EXT (516GG-983TT); INT (516GT-983TT or 516GG-983CT); and SLO (516TT-983TT, 516GT-983CT). b Composite 15582/516/983 were assigned as follows: EXT (15582CC-516GG-983TT or 15582CT-516GG-983TT); INT (15582TT-516GG-983TT, 15582CC-516GT-983TT, 15582CC-516GG-983CT, 15582CT-516GT-983TT, or 15582CT-516GG-983CT); and SLO (15582CC-516TT-983TT, 15582CC-516GT-983CT).

PLASMA EFAVIRENZ CONCENTRATIONS ARE ASSOCIATED WITH LIPID AND GLUCOSE CONCENTRATIONS

Sinxadi et al.

Supplementa1 Table 1. Multivariate linear regression analyses (each adjusted for age, body mass index, total duration on ART and current stavudine use) between $\log_{10}$ transformed efavirenz mid-dosing interval concentrations and each metabolic parameter (N=106)

Outcome variable	β (95%CI)	*Mean change (95% CI) per doubling of efavirenz concentrations (mmol/L)	p-value
Total cholesterol	1.31 (0.70 to 1.93)	0.39 (0.21 to 0.58)	0.000
LDL cholesterol	0.60 (0.12 to 1.08)	0.18 (0.04 to 0.32)	0.015
HDL cholesterol	0.45 (0.23 to 0.67)	0.14 (0.07 to 0.20)	0.000
Triglycerides	0.59 (0.09 to 1.09)	0.18 (0.03 to 0.33)	0.020
Fasting glucose	0.59 (0.08 to 1.09)	0.18 (0.02 to 0.33)	0.023
2-hour glucose	1.10 (0.21 to 1.99)	0.33 (0.06 to 0.60)	0.016

ART= antiretroviral therapy, N = total number of the study population, LDL= low-density lipoprotein, HDL = high-density lipoprotein. *Mean change (95%CI) in lipid and glucose concentrations (in mmol/L) with doubling of efavirenz concentrations. The mean changes are calculated using the formula Y= β*log10 (2), where β is the beta coefficient for the $\log_{10}$ transformed efavirenz concentrations as the independent variable in linear regression analyses for the various metabolic outcomes.
